# Do parenting practices moderate the association between the physical neighbourhood environment and changes in children’s time spent at various physical activity levels? An exploratory longitudinal study

**DOI:** 10.1186/s12889-021-10224-x

**Published:** 2021-01-19

**Authors:** Sanne M. P. L. Gerards, Dave H. H. Van Kann, Stef P. J. Kremers, Maria W. J. Jansen, Jessica S. Gubbels

**Affiliations:** 1 Department of Health Promotion, Nutrition and Translational Research Institute Maastricht (NUTRIM), Maastricht University, P.O. Box 616, Maastricht, MD 6200 The Netherlands; 2 School of Sport Studies, Fontys University of Applied Sciences, P.O. Box 347, Eindhoven, AH 5600 The Netherlands; 3 Department of Health Services Research, Care and Public Health Research Institute (CAPHRI), Maastricht University, P.O. Box 616, Maastricht, MD 6200 The Netherlands; 4 Academic Collaborative Centre for Public Health, Public Health Service South Limburg, P.O. Box 33, Heerlen, AA 6400 The Netherlands

**Keywords:** Children, Neighbourhood characteristics, Parenting practices, Physical activity, Physical environment, Sedentary behaviour

## Abstract

**Background:**

As many children do not meet the recommended daily physical activity (PA) levels, more research is needed towards environmental determinants of children’s PA levels. The aims of this longitudinal study were to investigate whether the physical environment and parenting practices have an impact on changes in children’s weekday time spent at various PA levels and whether associations between physical neighbourhood environment and changes in children’s PA are moderated by parenting practices.

**Methods:**

We performed a secondary data analysis of longitudinal data collected at three timepoints (baseline, 6, and 18 months) from 10 control schools of the Active Living study, a quasi-experimental study, which took place in South Limburg, the Netherlands. In total, 240 children aged 8–12 years were included in the analyses. PA levels were measured using accelerometry (ActiGraph GT3X+). The physical environment was assessed at baseline through neighbourhood audits of the school environment, and PA parenting practices were measured at baseline via validated parental questionnaires. Multivariate multilevel regression analyses were conducted to determine the main effects of the physical environment and parenting practices on changes in children’s time spent in sedentary behaviour (SB), light PA and moderate-to-vigorous PA (MVPA) over 18 months. Additionally, moderation of the association between the physical environment and children’s PA levels by parenting practices was examined by adding interacting terms to the regression equations.

**Results:**

Walkability of the physical environmental was associated with a decrease in SB at 18 months (B = -5.45, *p* < .05). In addition, the parenting practice logistic support was associated with an increase in MVPA (at all time points, B = .68, B = .73 and B = 1.02, respectively, all *p* < .05) and a decrease in SB (at 18 months, B = -1.71, *p <* .05). Stratified analyses (based on significant interaction terms) showed that the effect of specific physical environmental features (e.g., sports facilities) on children’s improvements in PA levels were strengthened by favourable parenting practices.

**Discussion:**

Besides the main effects of walkability and logistic support, there were indications that several parenting practices moderate the association between the physical environment and changes in children’s time in various PA levels. The current findings are exploratory, and need to be confirmed in further research.

## Background

Children are recommended to participate in moderate-to-vigorous intensity physical activity (MVPA) for at least 60 min per day [1]. However, children’s physical activity (PA) levels are insufficient worldwide [2]. According to the Netherlands’ Report Card on Physical Activity for Children and Youth [3], 72% of Dutch children do not meet the PA recommendations as proposed by the World Health Organization. Physical inactivity and sedentary behaviour of children have considerable health consequences, such as increased risk of obesity, metabolic syndrome and depression [4].

The time children spend in MVPA, light physical activity (LPA) and sedentary behaviour (SB) can partially be explained by factors in the environment, including the home, school and neighbourhood environment. Within these environments, different types can be distinguished, including the physical and social environment [5]. Observational studies increasingly show the importance of the physical environment on children’s PA. Multiple systematic literature reviews have been conducted to summarize the evidence regarding the role of the physical neighbourhood or the built environment on children’s PA. Ding et al. [6] published a systematic review on the association between the built environment and PA among children. They found consistent associations between environmental attributes, such as walkability, and children’s PA. Timperio et al. [7] also performed a systematic literature review regarding the role of the built environment on children’s PA. They found strong evidence that the walking and cycling infrastructure (for example presence and quality of sidewalks and bike paths or bike lanes) are most consistently correlated with PA in children [8–10].

Regarding children’s social environment, several systematic reviews have assessed parental influence on children’s PA [11–14], all underlining the influence of parental behaviours on children’s PA. Parenting practices are specific behaviours of parents influencing their child’s behaviour [15]. PA parenting practices are specific actions of parents in respect to their child’s physical activity. The impact of PA parenting practices on children’s PA is evident [16, 17]. A broad range of PA parenting practices can be distinguished, for example logistic support (providing funding, transport or equipment), providing a good example (modelling) and parental encouragement or policies (rules) [18, 19]. Prior research showed that supportive parenting practices (for example logistic support) are associated with more MVPA in children [20, 21]. Similarly, modelling and parental policies seem to be associated with objectively measured child PA [19, 21]. However, most previous studies on parenting practices used a cross-sectional design [16], which limits the possibility to make any causal conclusions.

Environmental determinants of behaviour should not be viewed in isolation. Based on socio-ecological frameworks, social environmental characteristics are presumed to interact with physical environmental characteristics in their influence on children’s behaviour [22]. The influence of a physical environment, whether supportive or unsupportive, on PA and SB has to be viewed in the context of the family environment, i.e. taking into account parenting practices [7]. There is growing evidence for the interaction between the social and physical environments on child PA. Ghekiere and colleagues [23] found that the positive effect of intersection density on the frequency of active trips was moderated by parent accompaniment, with the association only being present when parents were not accompanying their child. Furthermore, D’Haese and colleagues [24] found some indications for an interaction between the built environment and parenting practices in determining children’s MVPA. They found that parental MVPA was positively associated with child MVPA only when parents reported a high presence of sporting venues. An explanation can be that access to sporting venues may be an important prerequisite for parents to influence their children’s PA. Further insight into the interaction between the physical and social environment on children’s PA levels is needed, since to date, only a limited number of studies performed moderation analyses, and these were on only a limited number of interactions. Furthermore, the physical environment has not always been measured objectively [24] and conclusions often rely on cross-sectional designs [23, 24].

In the current exploratory study, we investigated the moderating effect of parenting practices on the association between the physical environment and changes in children’s weekday time spent at various PA levels, using a longitudinal design. For this purpose, we combined parent-reported parenting practices (questionnaire data) with objectively measured child PA data and data of school neighbourhood audits. Our hypothesis was that parenting practices can either buffer of exacerbate influences of the physical environment.

## Methods

### Setting and study design

We analysed data that were collected as part of the Active Living study [25], as secondary data-analysis. The design of this study is quasi-experimental with ten intervention and ten matched control schools in areas in South Limburg, the Netherlands. The Active Living project targets 6th – to 8th grade primary school children (8 to 12 years old). We visited all participating schools to inform the children about the study before data collection. All children attending grade 6 and 7 of the participating schools at baseline were eligible for participation in the study. Children who were interested in participating in the study received written information and a written informed consent form for their parents. Children were only included when they provided oral consent and when their parents provided written consent. Children were able to withdraw at any time from the study. Ethical approval for the Active Living study was obtained from the research ethics committee of the University Hospital Maastricht (METC 12–4-077). For more information about the Active Living study, we refer you to Van Kann et al. [25].

### Data collection and study population

In this study, we performed secondary analyses on the data from the 10 control schools of the Active Living study. We conducted measurements at different time points: baseline (T0), which took place between September and December 2012, and two follow-up measurements, which took place at 6 months (T1; between March and June 2013) and 18 months after baseline (T2; between March and June 2014). At baseline, we collected accelerometry, physical environment and parenting practices data, while at 6- and 18-months follow-up we only collected accelerometry data.

All participating schools were visited by two researchers and an employee of the Regional Public Health Service at the three measurement time points. Children received instructions for wearing the accelerometer and received a parental questionnaire, which they had to give to their parents to be returned to the school in a supplied envelope. One week later, a research assistant collected the accelerometer and the questionnaire.

In total, we approached 607 children to participate in the study (all children from the 6th – to 8th grade of the 10 participating schools). Of these, 331 (54.5%) received parental consent to wear an accelerometer. Of these 331 children, 251 parents (75.8%) filled in the parental questionnaire at baseline. Furthermore, 11 children were excluded at baseline because they did not fulfil the wear time validation criteria for the accelerometer. This resulted in 240 (39.5% of the 607) child-parent pairs at baseline. At post-test, respectively 212, and 191 children provided valid accelerometer data at 6 and 18-months follow-up. In total, 240, 202 and 189 children were able to be included in the analyses to predict PA at baseline, 6 and 18-months follow-up, respectively. These children provided valid data on PA and covariates. The main reason for drop-out was illness on the measurement day or having changed schools.

### Measures

#### Accelerometry

Children’s PA and SB levels were measured using accelerometers (ActiGraph, GT3X+, 30 Hz). During classroom visits, children were given an accelerometer. During these visits, we registered children’s gender and date of birth. We instructed children to wear a belt with the attached accelerometer on their right hip, for at least five consecutive days, including a weekend. We also instructed the children to remove the accelerometer only at night (while sleeping) or while they were swimming, bathing or taking a shower. Accelerometry data were processed using ActiLife version 6.10.4 (ActiGraph, Pensacola, USA). Choi and colleagues’ [26] wear time validation parameters were applied to the data and the minimal wear time per day was defined at 480 min (8 h) during waking hours (6 am-11 pm). To prevent reactivity to the measurement equipment, the first day of measurement was omitted [27]. Evenson’s cut-off values were used to determine SB (counts-per-minute (CPM) < 101), LPA (CPM = 101–2295) and MVPA (CPM > 2295) [28], as these cut-off points are validated for and widely used in studies concerning the current age group [29]. Time spent in SB, LPA and MVPA was divided by wear time to calculate a proportion of time per day spent at each activity level. Valid PA data during weekdays (minimum one wear day, excluding the first day of measurement) were aggregated into time spent in SB, LPA and MVPA per child. On average children provided valid PA data on two weekdays at all time points (T0: M = 1.89, SD = 0.32, range 1–2 days | T1: M = 1.93, SD = 0.48, range 1–3 days | T2: M = 1.98, SD = 0.69, range 1–4 days). Weekend days were not included in the analyses.

#### Physical environment

An environmental scan was conducted for all participating schools in August 2012, prior to the start of the Active Living project. The SPACE checklist [30] was used to assess physical neighbourhood characteristics. SPACE is a validated instrument based on the Neighborhood Environment Walkability Scale (NEWS [31]). As the relationship between environment and active transportation (walking and cycling) may be considered as either purpose-specific or country-specific, the NEWS was adapted to the Dutch situation. Additionally, modifications were based on outcomes of focus group interviews with children aged 6–11 years old leading to the final version of the SPACE checklist [30]. The final instrument consisted of 54 items measuring PA friendliness of neighbourhoods, assessing factors such as playground characteristics and general impression of the neighbourhood. Neighbourhood characteristics were assessed in an 800-m crow-fly buffer around the primary school the child attended. Schools were centralized and hypothesized to be the most commonly used physical environment for primary school children, as Dutch children tend to live quite close to their primary school [32]: the average distance between home and the nearest primary school in the Netherlands is 700 m [33]. Moreover, even the children who do not live within the 800-m buffer are very likely to be exposed to this environment daily when commuting to school. Observations were performed by two trained observers, with a substantial inter-rater reliability (Cohen’s Kappa = .73, *p* < .01). Both observers walked all street segments present in the 800 m buffer zone. They audited this school environment independently, but at the same time to prevent potential time-related bias, e.g., traffic density. It took about 4 h per school environment to complete the audit. We used the mean scores of the two auditors in the analyses.

Four measures of the audits were included in the current study. The *number of playgrounds* was summed as well as the *number of sports facilities* (i.e. sports centres and tennis courts). Furthermore, *walkability* was operationalized as an average score of the availability of sidewalks and walking short-cuts (routes for pedestrians which are shorter than the main streets, for example a footpath). Similarly, *cyclability* was operationalized as an average score of the availability of cycle paths and cycling short-cuts. The answer categories of the walkability and cyclability items were 4-point frequency scales ranging from none (1) to a few/a little (2), somewhat/little-to-many (3), and many (4).

Furthermore, weather conditions, i.e. temperature, rainfall and hours of sunshine, were obtained for every hour of all measurement days from the Royal Netherlands Meteorological Institute (KNMI) [34], as measurement season and thus weather conditions could vary accross measurements. Averaged weather condition data were calculated from the daily averages, over the valid PA days per child, resulting in child-specific weather condition data.

#### Social environment: parenting practices

Parenting practices were measured using a validated questionnaire developed and validated by Davison et al. [18], measuring parenting practices *logistic support* and *coactivities* (mentioned as ‘explicit modelling’ by Davison). The questions were translated into Dutch. Logistic support was measured using three items (Cronbach’s Alpha = .73). An example item is: ‘I often drive or take my child to places where he/she can be active (e.g., parks, playgrounds, sports, or practices)’. Coactivities consisted of three items (Cronbach’s Alpha = .74), for example ‘I frequently exercise or do something active with my child’.

Furthermore, we used the validated instrument of Gattshall and colleagues [19], which measures *modelling* and *encouragement* (called ‘policies’ by Gattshall). Modelling was measured using four items (Cronbach’s Alpha = .62). An example item is: ‘Your child sees you being physically active’. Encouragement was measured using four items (Cronbach’s Alpha = .79), for example: ‘How often do you encourage your child to be physically active?’. All scales were measured using a 5-point frequency scale ranging from ‘never’ to ‘very often’.

#### Background variables

Children reported their age (date of birth) and gender (boy or girl). Other background variables were parent-reported in the parental questionnaire: relation to the child (father or mother), parents’ country of birth (recoded as the Netherlands or other), weight and height in order to calculate their Body Mass Index (BMI: weight (kg) / (height (m))^2^), and educational level. Educational level was recoded into low (lower vocational education), medium (from secondary vocational school to high school) and high (higher professional education or university education).

### Statistical analyses

Data were analysed using SPSS version 21.0 (IBM, USA). *P* < 0.05 was considered statistically significant. Means and frequencies of background variables and all outcome measures were calculated using descriptive statistics. Multilevel regression analyses were conducted to determine the main baseline effect of the physical neighbourhood environment (number of playgrounds, sports facilities, walkability and cyclability) and parenting practices (logistic support, modelling, coactivities and encouragement) on children’s PA levels (SB, LPA, and MVPA, adjusted for wear time) at baseline, 6-months, and 18-months follow-up. These models were adjusted for the multilevel structure of the data, comprising nesting of children within schools, including a random intercept for school in each of the models. We did not adjust for nesting of children within families, as there were no children from the same families that participated in the study. Each of the multilevel regression analyses was corrected for baseline PA level (by adding the baseline PA levels in the model, except for the models with baseline PA as the dependent variable) and potentially relevant confounders (child age and gender, and weather conditions: temperature, rainfall and hours of sun), which were tested using backward deletion. We tested all the characteristics in one model in which the physical environmental characteristics and the parenting practices were combined.

We then explored whether parenting practices moderated the association between physical neighbourhood environment variables and children’s PA levels (SB, LPA, MVPA on baseline, 6-months and 18-months follow-up) by adding the interaction terms between each of the parenting practices and each physical environmental factor one by one to the model. This was done for each outcome variable, resulting in a total of 144 interaction terms being tested. The threshold for including interaction terms was set at *p* < 0.10, as suggested by Stone-Romero and Liakhovitski [35]. In the case of a significant interaction term, stratification by the concerning parenting practice was performed based on median split for the parenting practice: low < 4 and high ≥4 for all practices. The multilevel analysis was then repeated separately for the low and high strata of the parenting practice, omitting the parenting practice and interaction term from the model. Again, all these stratified regression analyses were corrected for baseline PA levels and relevant significant confounders (child age and gender, and weather conditions: temperature, rainfall and hours of sun), which were tested using backward deletion.

## Results

At baseline, children participating in the study were 9.74 years old (standard deviation (SD): .73), and there was a slightly higher proportion of boys (55.2%) compared to girls (see Table 1 for the general characteristics of the study population). Most participating parents were mothers, had a medium educational level and were native Dutch. The average age of the parents was 41 years old (SD: 4.58) and their mean BMI was 24.74 (SD: 3.56).

**Table 1:** General characteristics of the participants (*N* = 240)

	N	(%)	Mean	SD
*Characteristics of parents*				
Participant				
Mother	200	83.3		
Father	40	16.7		
Age^a^			41.47	4.58
Educational level				
Low	36	15.1		
Medium	109	45.8		
High	93	39.1		
Parents’ country of birth				
Both NL	221	92.9		
Other	17	7.1		
BMI of parents			24.73	3.56
*Characteristics of the child*				
Gender				
Girl	107	44.8		
Boy	132	55.2		
Age^a^			9.74	.73
Grade				
6th	117	49.4		
7th	119	50.4		

Numbers differ due to missing data; ^a^ age is measured in years

Children spent on average more than 8 h (> 500 min per day) in SB during waking hours, at every time point (baseline, 6 months and 18 months). Moreover, on average they spent more than 50 min on MVPA and more than 195 min on LPA daily (see Table 2 for means and standard deviations). Respectively 29.2, 42.0, and 35.1% of children achieved at least 60 min of MVPA at baseline, 6-months and 18-months follow-up.

**Table 2:** Means and standard deviations of parenting practices, physical neighbourhood environment characteristics and time children spent in PA levels

		Mean ± SD	
**Parenting practices**	Logistic support^a^	4.12 ± .63	
	Coactivities^a^	3.21 ± .76	
	Modelling^a^	3.48 ± .62	
	Encouragement^a^	3.84 ± .67	
**Neighbourhood characteristics**	Number of playgrounds	9.48 ± 4.23	
	Number of sports facilities	2.02 ± 1.33	
	Walkability^b^	3.45 ± .23	
	Cyclability^b^	2.96 ± .69	
		Mean minutes ± SD	Mean valid days ± SD
**Valid wear time**	Baseline	781.74 ± 80.75	1.89 ± 0.32
	6-Months follow-up	781.72 ± 92.70	1.93 ± 0.48
	18-Months follow-up	768.12 ± 98.41	1.98 ± 0.69
		Mean minutes ± SD	Mean % per day ± SD
**Child PA levels**	LPA baseline	212.07 ± 39.43	27.21 ± 4.65
	LPA 6-months follow-up	217.05 ± 44.26	27.81 ± 4.86
	LPA 18-months follow-up	195.01 ± 43.73	25.39 ± 4.94
	MVPA baseline	50.63 ± 21.62	6.49 ± 2.69
	MVPA 6-months follow-up	56.57 ± 24.18	7.27 ± 3.08
	MVPA 18-months follow-up	54.32 ± 25.99	7.07 ± 3.28
	SB baseline	519.03 ± 76.71	66.30 ± 6.03
	SB 6-months follow-up	507.97 ± 81.56	64.92 ± 6.42
	SB 18-months follow-up	518.81 ± 84.35	67.54 ± 6.76

*SB* Sedentary Behaviour, *LPA* Light Physical Activity, *MVPA* Moderate-to-Vigorous Physical Activity. ^a^ scale 1–5, ^b^ scale 1–4

### The association between physical environment and parenting practices on PA levels (main effects)

In the multilevel multivariate regression models testing the effect of physical environmental characteristics and parenting practices on children’s time spent at the various PA levels (Table 3), we found that walkability was associated with a decrease in proportion of time spent in SB at 18-months follow-up. Furthermore, we found that logistic support was associated with an increase in proportion of time spent in MVPA over time and a decrease of proportion of time spent in SB at 18-months follow-up. There were no significant associations between any of the other practices or neighbourhood environmental factors and the PA outcomes.

**Table 3:** Regression coefficients of combining physical environment and parenting practices on proportion of time in PA levels

Unstandardized Regression Coefficients (95%CI)									
	LPA T0	LPA T1	LPA T2	MVPA T0	MVPA T1	MVPA T2	SB T0	SB T1	SB T2
**Total model**									
Logistic support	.339 (−.680; 1.338)	.381 (−.675; 1.438)	.721 (−.403; 1.845)	**.684 (.096; 1.272)**	**.725 (.108; 1.343)**	**1.023 (.309; 1.738)**	−.983 (− 2.274; .3997)	− 1.283 (−2.624; .058)	**− 1.713 (−3.214; −.212)**
Coactivities	.639 (−.345; 1.622)	−.710 (− 1.758; .338)	.318 (−.805; 1.441)	−.119 (−.688; .450)	.072 (−.524; .668)	−.052 (−.743; .640)	−.638 (− 1.933; .656)	.613 (−.705; 1.931)	−.276 (− 1.753; 1.201)
Modelling	−1.080 (− 2.296; .136)	.559 (−.734; 1.852)	−.372 (− 1.738; .994)	−.369 (1.063; .326)	−.622 (− 1.359; .114)	−.543 (− 1.384; .298)	1.475 (−.112; 3.063)	.208 (−1.426; 1.841)	.851 (−.962; 2.664)
Encouragement	.022 (−.946; .990)	.256 (−.726; 1.237)	.139 (−.903; 1.180)	.255 (−.303; .814)	−.081 (−.644; .482)	−.473 (− 1.121; .175)	−.247 (− 1.517; 1.022)	−.211 (− 1.449; 1.026)	.296 (1.086; 1.678)
N playgrounds	−.192 (−.751; .368)	−.192 (−.666; .281)	.139 (−.293; .571)	−.112 (−.375; .151)	−.154 (−.564; .255)	−.037 (−.314; .241)	−.033 (−.791; .724)	.243 (−.525; 1.010)	−.096 (−.588; .395)
N sports facilities	−.293 (− 1.361; .776)	.271 (−.690; 1.232)	−.198 (− 1.012; .616)	−.110 (−.637; .416)	.199 (−.553; .950)	−.014 (−.585; .556)	−.027 (− 1.556; 1.502)	−.092 (− 1.645; 1.461)	.325 (−.706; 1.355)
Walkability	−.502 (−6.597; 5.594)	3.845 (.1.525; 9.216)	2.632 (−1.195; 6.458)	1.136 (− 1.865; 4.136)	3.224 (− 2.306; 8.755)	1.873 (− 1.403; 5.149)	−.258 (− 8.863; 8.348)	−5.744 (− 14.463; 2.976)	**−5.451 (− 10.273; −.629)**
Cyclability	.228 (− 2.458; 2.913)	.677 (−1.589; 2.944)	− 1.607 (− 3.512; .298)	−.053 (− 1.293; 1.188)	−.860 (− 2.818; 1.098)	.677 (−.677; 2.032)	.810 (− 2.794; 4.414)	−.543 (− 4.201; 3.116)	1.075 (−1.160; 3.310)

All analyses were corrected for relevant covariates tested with backward deletion. Models predicting PA level on T1 and T2 were also corrected for baseline PA level, *LPA* Light Physical Activity, *MVPA* Moderate-to-Vigorous Physical Activity, *SB* Sedentary Behaviour, *T0* baseline, *T1* 6-months follow-up, *T2* 18-months follow-up, bold numbers are statistically significant (*P <* 0.05)

### Exploration of the moderating effect of parenting practices on the association between physical environment on PA level

**Fig. 1:**
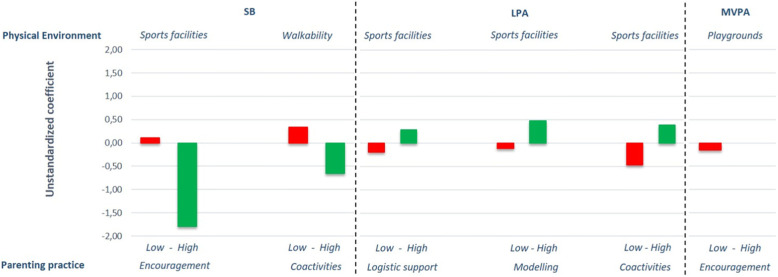
The influence of the physical environment on SB, LPA, MVPA at baseline for significant parenting practices stratified for high and low scores on parenting practices. *Note: Stratified analyses based on significant interaction terms in the full sample. All analyses were corrected for relevant covariates tested with backward deletion. None of the presented stratified effects was statistically significant. SB = Sedentary Behaviour; LPA = Light Physical Activity; MVPA = Moderate-to-Vigorous Physical Activity. Green bars represent desirable effects; red bars represent undesirable effects*

**Fig. 2:**
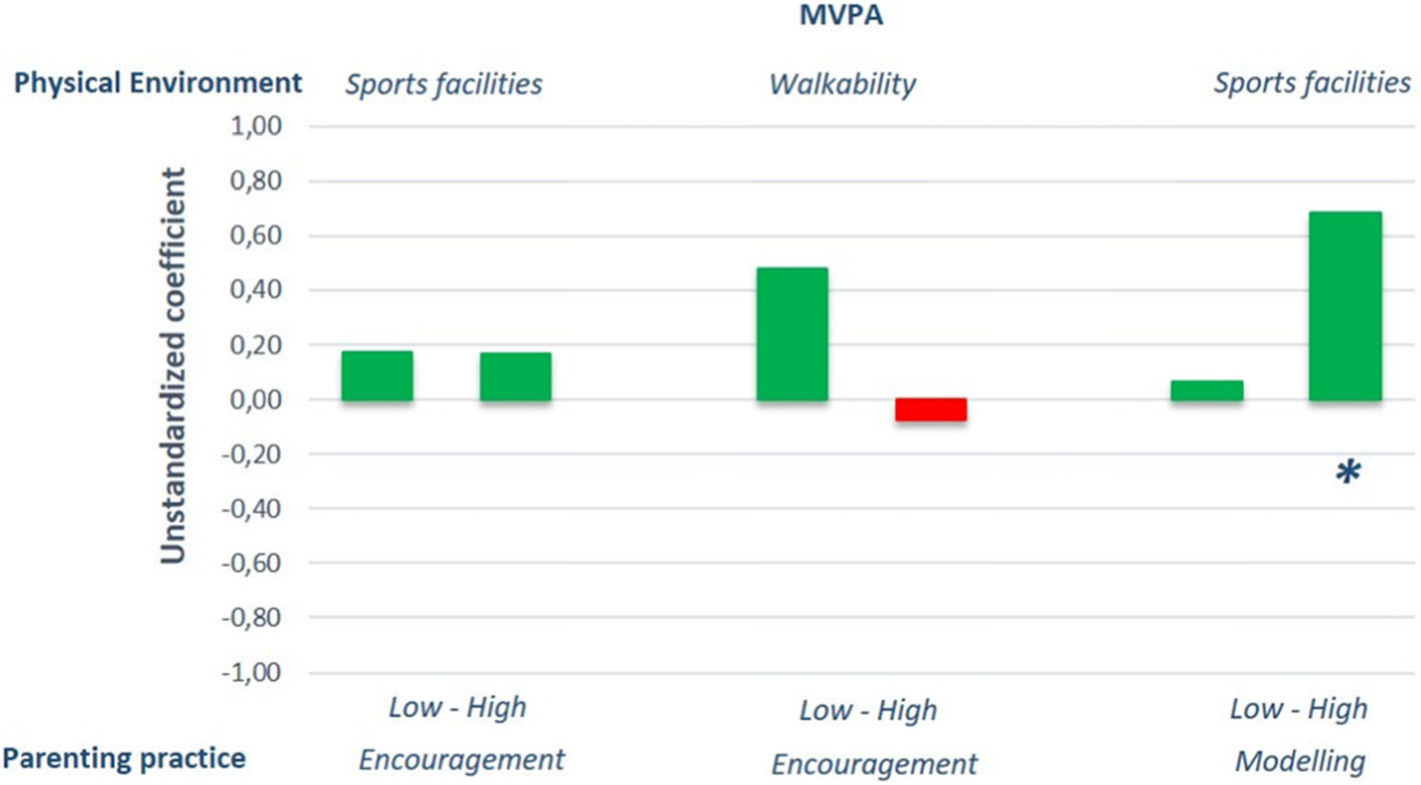
The influence of the physical environment on MVPA at 6-months follow-up for significant parenting practices stratified for high and low scores on parenting practices. Note: Stratified analyses based on significant interaction terms in the full sample. All analyses were corrected for relevant covariates tested with backward deletion and baseline MVPA levels respectively. * = p < .05; MVPA = Moderate-to-Vigorous Physical Activity. Green bars represent desirable effects; red bars represent undesirable effects

**Fig. 3:**
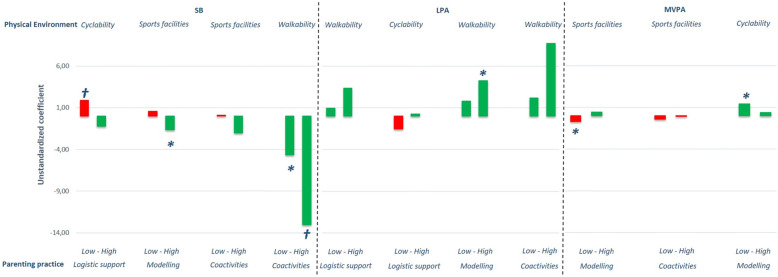
The influence of the physical environment on SB, LPA, MVPA at 18-months follow-up for significant parenting practices stratified for high and low parenting practices. Note: Stratified analyses based on significant interaction terms in the full sample. All analyses were corrected for relevant covariates tested with backward deletion and baseline SB/LPA/MVPA levels respectively. * = p < .05; † = p < .10; SB = Sedentary Behaviour; LPA = Light Physical Activity; MVPA = Moderate-to-Vigorous Physical Activity. Green bars represent desirable effects; red bars represent undesirable effects

Of all the interaction effects we explored in the full sample, we found that 20 interaction effects (14%) were significant (*p* < .10; data not presented). For these 20 interaction effects, subgroup analyses (based on a median split) showed that there were clear trends in the data (see Figs. 1, 2 and 3 depicting subgroup analyses for significant interaction effects only). Although most subgroup effects did not reach statistical significance, the positive associations of the physical environment (e.g., sports facilities, walkability, cyclability and number of playgrounds) with change in children’s time spent at certain PA levels were, quite consistently, stronger when their parents had favourable parenting practices (e.g., higher scores on coactivities and modelling), while unfavourable parenting practices (i.e. lower scores on the parenting practices) weakened positive neighbourhood effects (shorter green bars in Figs. 1, 2 and 3), or even resulted in counterproductive effects (red bars in Figs. 1, 2 and 3).

To illustrate such counterproductive effects, the availability of sports facilities had no significant effect on children’s proportion of time spent in MVPA when their parents scored high on modelling (B = 0.41, *p* > .10) and a significant negative effect on children’s proportion of time spent in MVPA at 18-months follow-up when their parents scored low on modelling (B = − 0.71, *p* < .05). Similarly, cyclability was not significantly associated with children’s proportion of time spent in SB when parents scored high on logistic support (B = − 1.21, *p >* .10) and positively related to children’s proportion of time spent in SB at 18-months follow-up when parents scored low on logistic support (B = 1.89, *p* < .10). Both examples indicate a counterproductive effect of the physical environment when parents either did not model PA or were not supportive for the child’s PA (see Fig. 3).

An example of a physical environmental association being strengthened by a positive parenting practice is for instance the association between sports facilities and MVPA at 6 months follow-up (see Fig. 2). While there was a significant and positive association when parental modelling of PA was high (B = 0.69, *p* < .05), the association was non-significant if parental modelling was low (B = 0.06, *p* > .10). Similarly, walkability was significantly positively associated with LPA at 18 months follow-up if parental modelling was high (B = 4.27, *p <* .05), but non-significant when parental modelling was low (B = 1.82, *p >* .10; see Fig. 3). In line with these examples, all subgroup analyses except for two showed such strengthening effects of positive parenting practices on the impact of positive physical environmental characteristics on children’s PA levels. One exception was the moderation of encouragement on the association between walkability on MVPA at 6-months follow-up (stronger association when parents offered low encouragement, see Fig. 2). The other exception was the moderation of modelling on the association between cyclability and children’s MVPA level at 18-months follow-up (stronger association when parents offered low modelling, see Fig. 3).

## Discussion

The aim of the current longitudinal study was to explore whether PA parenting practices moderated the influence of the physical neighbourhood characteristics on children’s PA levels. It was one of the first studies on this topic that combined a longitudinal design with objective outcome measures, in which physical environment was systematically assessed and validated parenting instruments were used. We found that some characteristics of both the social and physical environment were significant predictors of changes in children’s time in PA and SB. Additionally, the explorative moderation analyses showed that several parenting practices moderated the impact of some physical neighbourhood characteristics on children’s PA levels in such a way that positive parenting practices strengthened positive influence of a PA-promoting neighbourhood, while less positive practices diminished this positive neighbourhood influence or even led to undesirable effects.

As regards the influence of the physical neighbourhood environment on children’s PA levels, we found that walkability was significantly negatively associated with children’s SB at 18-months follow-up. This is in line with prior research, as various systematic reviews indicate walking infrastructure as a consistent positive predictor of children’s PA [6, 7, 36]. Unexpectedly, the number of playgrounds in the neighbourhood was not significantly associated with children’s time spent in the various activity levels, whereas most studies found a positive influence of playground density on children’s PA ([37]).

Concerning the parenting practices, we found that logistic support was a significant positive predictor of the change in child’s time in MVPA up to 6 months after baseline, and a significant negative predictor of children’s time in SB (i.e. less time spent in SB) at 18 months. Children whose parents provided more logistic support at a younger age spent about five additional minutes in MVPA per day 18 months later, which represents almost 10% of the WHO recommendation for daily MVPA. This finding confirmed earlier research of O’Connor [20], who also found an association of logistic support with children’s PA level. We did not find an association between parental modelling and children’s PA, which is also in line with the findings of O’Connor et al. [20]. Gattshall and colleagues [19], however, did find an effect of role modelling and encouragement on overweight children’s PA level. Previous research has indicated that effects of parenting practices are moderated by child weight status [38], potentially explaining the differences with Gattshall’s findings. Moderation by weight status was, however, not examined in the current study.

Interestingly, MVPA increased with age in our sample: children were more active at both follow-up moments, compared to baseline PA. This contradicts previous findings that children generally become less active with increasing age [39, 40]. It is assumed that our findings reflect a seasonal effect (e.g., amount of daylight), despite correcting for weather conditions, as both follow-up measurements were conducted in spring, while the baseline measurement was done in autumn.

Although most interactions were non-significant, there were some indications that effects of the physical environment on children’s PA levels were moderated by PA parenting practices (14% of the 144 tested interactions), which is in line with the social-ecological perspective [22]. This indicates that by interpreting only the main effects, we could potentially overlook important information. The majority of the interaction effects found (18 out of 20), were in the expected direction: the effect of the physical neighbourhood environment on child’s PA level was strengthened by supportive parenting practices. The combination of supportive parenting and a PA-promoting neighbourhood may thus lead to synergistic effects, as hypothesized in ecological models [22]. Parents seem to act as gatekeepers to the potential positive effects of the neighbourhood, which seems logical: a child can only be influenced by his or her neighbourhood if a parent allows and supports the child to actually utilize this neighbourhood [41]. Along this line, negative parenting practices (i.e. psychological control to decrease PA) have previously been reported to diminish the positive effect of outdoor location on children’s PA [42]. A study among adults from deprived neighbourhoods revealed a similar pattern, with physical environmental features influencing PA only when the social environment was positive [43]. However, we also found some indications that some PA-promoting neighbourhood characteristics were associated with counterproductive effects in the context of unsupportive parenting practices (e.g., a negative association between sports facilities and MVPA in the context of lack of parental modelling of PA). It is unclear why this is the case: more research will be needed to further examine this unexpected finding. Interestingly, Ghekiere and colleagues [23] found an opposite interaction effect: while parental co-participation in cycling diminished the effects of neighbourhood characteristics in their study, stronger effects were observed when parents co-participated in activities in the current study. The difference might be explained by the fact that our measurement of coactivities regarded general co-participation in physical activity, while Ghekiere et al. measured co-participation specifically on trips in the neighbourhood [23]. Furthermore, it should be noted that the majority of the tested interactions was not significant, and our study might have been underpowered for examining stratified associations. In view of this, our findings should be regarded as exploratory and interpreted cautiously, yet might help form hypotheses on interactions between environmental factors. More research will be needed to further examine and confirm these findings.

Generally, our findings underline the necessity of incorporating interactions between different types of environmental determinants in order to truly understand their effect on behaviour [22]. In line with the ecological view on (health) behaviour, we found that children may respond differently in the physical neighbourhood environment depending on parental cues (parenting practices) [44]. These two different interaction micro-systems (neighbourhood and home), can be recognized as a meso-system [45]. However, practice is doubtlessly even much more complex than our current understanding of environmental influences and the interactions found. Most likely, the interacting environmental influences examined in the current study (i.e., the meso-system of the physical neighbourhood environment and parenting practices) interact with other environmental factors (e.g., teacher practices, peer influences) and individual characteristics (e.g., gender, age, temperament) as well, introducing three-way and even more complex interactions [46]. The challenge for future research will be to design studies and analyses that are equipped and powered to examine such complex interaction between all factors involved [22].

A strong point of the current study is that we used a longitudinal design with 18-month follow-up. Moreover, we combined three different validated instruments, i.e. validated self-report questionnaires for parenting practices, neighbourhood audits, and objectively assessed PA data. Although we used validated instruments in the current study [18, 19], it should be noted that not all Dutch translations of the questionnaires were validated. In addition, the current instruments do not cover the complete spectrum of parenting practices [16]. In addition, our findings are not generalizable to the general population. Although we conducted the study in deprived areas, our sample was relatively highly educated, which is an indication of selection bias. It should further be noted that the response of fathers was rather low (16.7%). The sample sizes for some of the stratified analyses was further limited (*N* = 43), limiting the power of the analyses, hence the exploratory nature of our study. In addition, there was some attrition between the measures, potentially introducing bias. As the study was of exploratory nature, the results were not adjusted for multiple testing effects. With adjustment for multiple testing, a number of the reported associations would no longer be significant. The findings should thus be interpreted with caution. In assessing the physical environment, we used the validated SPACE checklist [30] in an 800-m buffer around school, with two independent researchers who performed the observations, with an acceptable inter-reliability score. It should be noted that in the Netherlands, 60–80% of the children live in this 800-m buffer [32], indicating that we could not capture the complete neighbourhood environment of all families in our sample. Although this is a limitation, the average distance between home and the nearest primary school in the Netherlands is 700 m [33]. Moreover, even the children who do not live within this 800-m buffer are very likely to be exposed to this environment daily when commuting to school. Also, the variation of the scores resulting from the audit is limited, both due to the relatively small number of schools (*n* = 10) and the limited variation in walkability and cyclability of neighbourhoods as Dutch residential areas tend to have excellent access to these infrastructural characteristics. Moreover, the walkability and cyclability scales were not validated. Another point to consider is that, although we corrected all the analyses for weather influences, seasonal effects might impact children’s PA levels. While this might explain the increase in MVPA between baseline and 6-months follow-up, these seasonal effects are not expected to influence the study objectives, i.e. the correlation between the physical environment, parenting practices and PA. Further, a minimum of one valid measurement day of accelerometer data was used, which might be insufficient for reflecting habitual PA. Future studies should plan on a greater number of accelerometer wear days to facilitate greater number of valid days for analysis (e.g., [47]). A final limitation is that only valid PA data on weekdays was available, since children did not wear the accelerometers consistently during the weekends. However, the inclusion of weekdays ensures exposure to the assessed neighbourhood characteristics as children go to school on weekdays, while exposure to the neighbourhood is less certain during the weekend. As such, focusing on weekdays only was considered justified. Nonetheless, the findings should be interpreted in light of these considerations.

Health promotion professionals should be aware of the moderating effect of parenting practices (social environment) on the physical environment, indicating that lack of attention for parenting practices can nullify investments in neighbourhood adjustments. On the other hand, positive parenting practices can strengthen neighbourhood effects. The effect of changes in the physical environment on children’s behaviour is thus dependent on the social environment, in line with previous studies examining such interactions. When financial resources exist, interventions should be applied in both environments in order to optimize intervention effectiveness. The synergetic effect of combining multiple types of environment needs to be acknowledged in practice to improve the effectiveness of PA interventions.

It is also important to realize that the effect of different parenting practices depends on the broader environment. Although logistic support was found to positively predict children’s PA levels 18 months later, the numerous interaction effects between parenting practices and the physical environment indicate that providing one universal message to parents seems to be suboptimal. If supportive practices cannot (sufficiently) be executed in the living environment, their effectiveness might be very limited. Similarly, health promotors should question whether interventions that target only parenting practices make sense in low SES areas, which are often the areas in which the physical environments are less supportive [48].

## Conclusions

Both the physical environment and the social environment (parenting practices) have an impact on children’s PA and SB. There were some indications that several parenting practices moderate the longitudinal association of the physical environment with change in children’s time spent at various PA levels (up to 18 months), emphasizing the need to take ecological interactions into account while explaining and affecting children’s PA. More research is needed to confirm the explorative findings.

## Data Availability

The datasets used and/or analysed during the current study are available from the corresponding author on reasonable request. Permission to access the data was obtained by D.H.H.V.K. and M.W.J.J.

## Abbreviations

PA: Physical Activity
SB: Sedentary Behaviour
LPA: Light Physical Activity
MVPA: Moderate-to-Vigorous Physical Activity
CPM: Counts-Per-Minute
NEWS: Neighbourhood Environment Walkability Scale
BMI: Body Mass Index
